# The potential role of the orexin system in premenstrual syndrome

**DOI:** 10.3389/fendo.2023.1266806

**Published:** 2024-01-16

**Authors:** Ping Dong, Weibo Dai, Mengyue Su, Shukun Wang, Yuexiang Ma, Tingting Zhao, Feng Zheng, Peng Sun

**Affiliations:** ^1^ School of Traditional Chinese Medicine, Shandong University of Traditional Chinese Medicine, Jinan, China; ^2^ Department of Pharmacy, Zhongshan Hospital of Traditional Chinese Medicine, Zhong Shan, China; ^3^ School of Pharmacy, Shandong University of Traditional Chinese Medicine, Jinan, China; ^4^ College of Foreign Languages, Shandong University of Traditional Chinese Medicine, Jinan, China; ^5^ Department of Neurosurgery, The Second Affiliated Hospital of Fujian Medical University, Quanzhou, China; ^6^ Innovation Research Institute of Chinese Medicine, Shandong University of Traditional Chinese Medicine, Jinan, China

**Keywords:** premenstrual syndrome, orexin, symptoms, mechanism, new target

## Abstract

Premenstrual syndrome (PMS) occurs recurrently during the luteal phase of a woman’s menstrual cycle and disappears after menstruation ends. It is characterized by abnormal changes in both the body and mood, and in certain cases, severe disruptions in daily life and even suicidal tendencies. Current drugs for treating PMS, such as selective serotonin reuptake inhibitors, do not yield satisfactory results. Orexin, a neuropeptide produced in the lateral hypothalamus, is garnering attention in the treatment of neurological disorders and is believed to modulate the symptoms of PMS. This paper reviews the advancements in research on sleep disturbances, mood changes, and cognitive impairment caused by PMS, and suggests potential pathways for orexin to address these symptoms. Furthermore, it delves into the role of orexin in the molecular mechanisms underlying PMS. Orexin regulates steroid hormones, and the cyclic fluctuations of estrogen and progesterone play a crucial role in the pathogenesis of PMS. Additionally, orexin also modulates the gamma-aminobutyric acid (GABA) system and the inflammatory response involved in coordinating the mechanism of PMS. Unraveling the role of orexin in the pathogenesis of PMS will not only aid in understanding the etiology of PMS but also hold implications for orexin as a novel target for treating PMS.

## 1 Introduction

**Figure 1:**
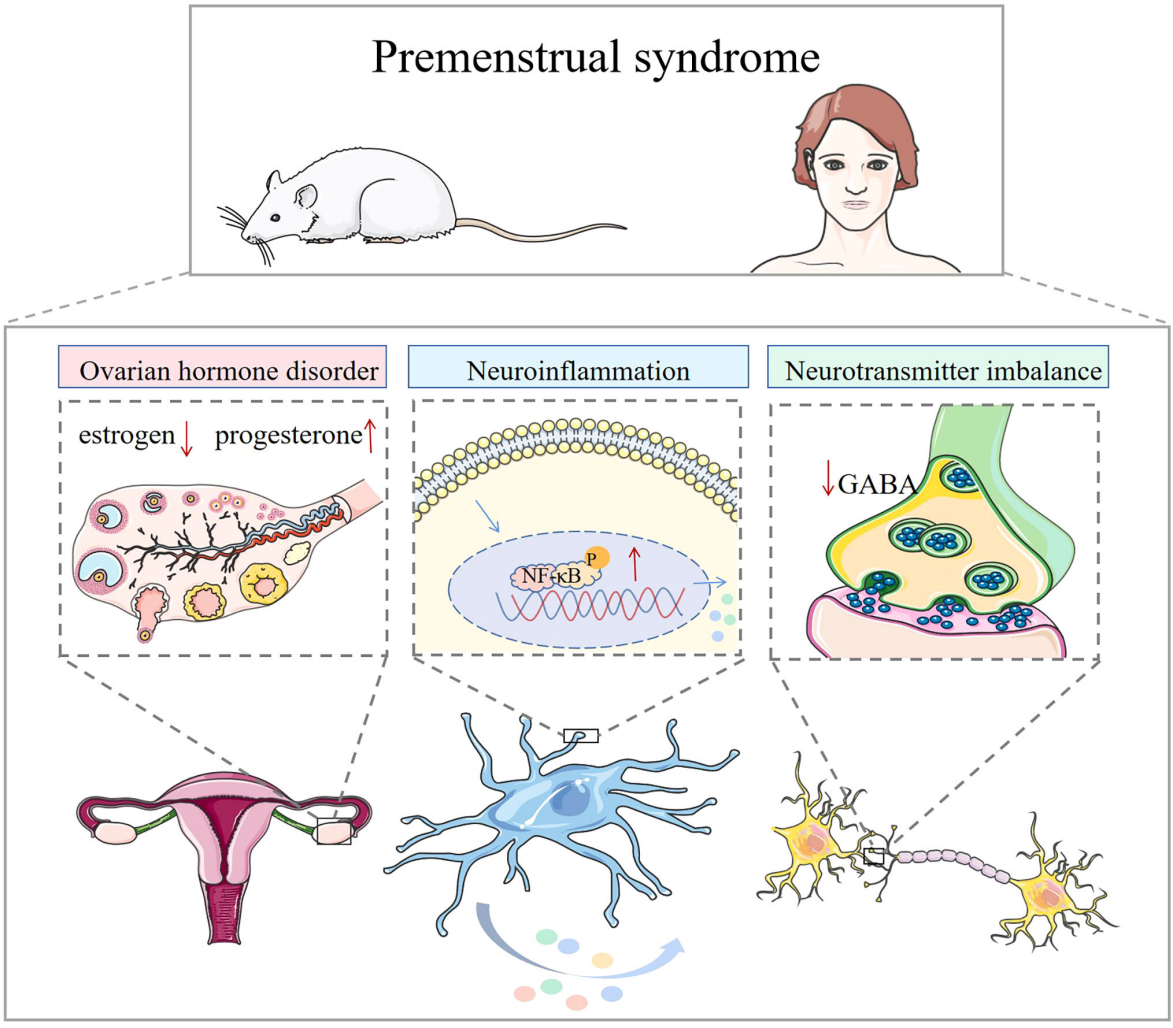
Mechanisms associated with premenstrual syndrome. In animals and humans, ovarian hormone disruption, elevated inflammatory factors, and neurotransmitter imbalance are associated with the development of premenstrual syndrome.

PMS is a common issue among women of childbearing age. The American College of Obstetricians and Gynecologists (ACOG), in its guidelines for women’s health, defines PMS as the cyclical onset of affective and somatic symptoms that occur 7 to 14 days before menstruation (luteal phase). Affective symptoms include anxiety, confusion, depression, irritability, and social withdrawal, while somatic symptoms encompass breast tenderness or swelling, headaches, joint or muscle pain, swelling of the extremities, and weight gain, with at least one affective and one somatic symptom (1, 2). Premenstrual dysphoric disorder (PMDD) represents a severe form of PMS. According to the Diagnostic and Statistical Manual of Mental Disorders (DSM), 3-8% of women of childbearing age are diagnosed with PMDD (3). These patients exhibit symptoms such as significant mood swings, subjective difficulties in concentrating, obvious changes in appetite, lethargy, or insomnia. Moreover, their conditions may exacerbate the progression of disorders like major depression, panic disorder, and personality disorders, significantly impacting work, studies, daily social activities, and relationships with others (4, 5). Therefore, it becomes increasingly important to gain a clearer understanding of the neurological aspects of premenstrual depression. Modern medical research has yet to establish a clear and consistent definition of the pathogenesis of PMS. Most experts believe it arises from the interaction of several factors (6), including dysregulation of hormone levels, especially estrogen and progesterone, which forms the primary theory for the development of PMS. Neurotransmitters such as 5-hydroxytryptamine (5-HT), dopamine, glutamate, noradrenaline, and GABA impact the psychological manifestations of the syndrome (7, 8). Abnormal responses to inflammatory factors may also be associated with disease development (9) (**Figure 1**).

**Figure 2:**
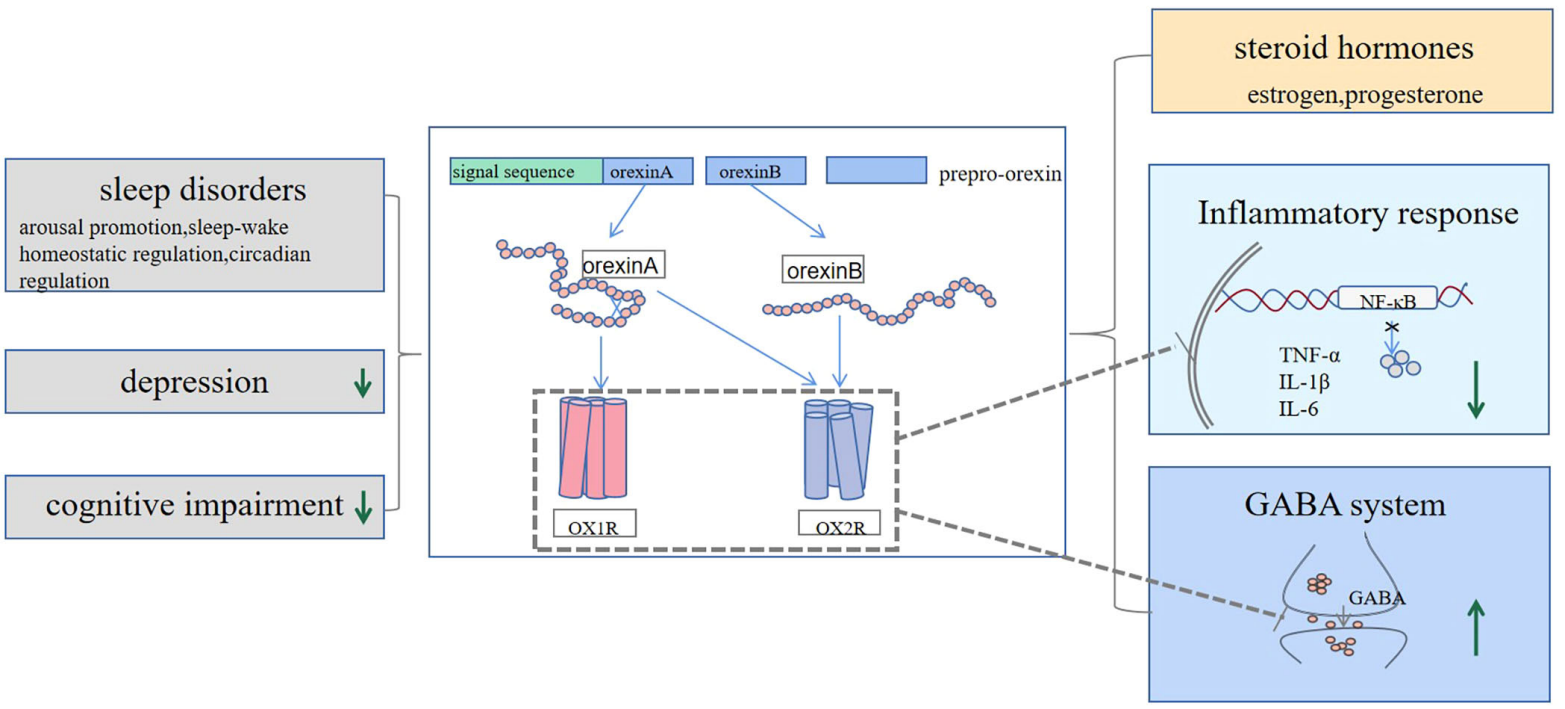
Orexin may have a potential therapeutic effect on PMS symptoms by reducing daytime sleepiness and increasing the duration of rapid eye movement sleep, improving cognitive function, and alleviating depressive mood. Mechanistically, orexin can modulate ovarian hormones, suppress elevated levels of inflammatory factors, and increase the release of GABA neurotransmitters, all of which may contribute to its positive effect on PMS symptoms.

Orexin, also known as hypocretin, derives its name from the Greek word “orexin”, which means ‘appetite’. Sakurai et al. named these neuropeptides ‘orexin’ due to their propensity to promote feeding (10). Based on their similarity to incretins “hypocretin”, de Lecea et al., in 1998 named them “hypocretins” (11). Studies have shown that prepro-hypocretin mRNA is exclusively expressed by neurons in the lateral hypothalamic area (LHA), including the lateral nucleus, posterior nucleus, and dorsomedial nucleus of the hypothalamus (12). Orexin-A and orexin-B are produced from prepro-orexin by enzymatic cleavage, with the former consisting of 33 amino acid residues and two intramolecular disulfide bridges in its N-terminal structural domain, and the latter comprising 28 amino acid residues. Orexin-A and orexin-B share 46% sequence homology (13). Orexin peptides are highly conserved in vertebrates, particularly in mammals: orexin-A has 100% sequence homology, while orexin-B differs by only 1 or 2 amino acids between species (12). Orexin-A exhibits equally high affinity for orexin receptor 1 (OX1R) and orexin receptor 2 (OX2R), while orexin-B demonstrates approximately 10-fold selectivity for OX2R. The orexin neuropeptide system has emerged as a potential new drug target for the treatment of psychiatric disorders (14). Despite orexin’s primary function being the maintenance of wakefulness, animal studies have revealed its influence on emotional, behavioral, and cognitive regulation, encompassing stress response, reward processing, sleep-wake regulation, and feeding (15, 16). This paper examines the relationship between premenstrual syndrome and orexin considering both clinical features and molecular biological mechanisms (**Figure 2**). This review may establish a theoretical basis for orexin-targeted therapy for PMS/PMDD.

## 2 Orexin reduced PMS-induced sleep disorders

Sleep disturbances in patients with PMS are typically characterized by subjective sleep disruptions, abnormal sleep electroencephalogram (EEG) patterns, and shifts in sleep-wake rhythm (17–19). In clinical studies, patients with PMS self-reported difficulties with nocturnal awakening and morning fatigue (20), often accompanied by varying levels of sleep deprivation (21). Some patients reported experiencing insomnia, inattention, and fatigue, with greater severity observed in women with PMDD compared to healthy women, especially during the luteal phase as opposed to the follicular phase (22). Conzatti et al. (23) observed in their study that 49.3% of PMS patients experienced excessive daytime sleepiness (EDS) during the luteal phase, with the likelihood of EDS during the follicular phase at 48.8%. In an epidemiological survey, women with PMS exhibited an increased total sleep time as recorded by polysomnography (PSG) and lower peripheral oxygen saturation levels (24). Women with severe PMS also showed significantly increased latency to rapid eye movement (REM) sleep compared to those with no or mild PMS. Additionally, detailed analysis of the sleep EEG revealed subtle differences; when compared with asymptomatic control subjects, women with PMDD demonstrated decreased occurrence of delta waves and increased occurrence and amplitude of theta waveforms in non-REM sleep (25). Furthermore, changes in the circadian rhythm of sleep during the menstrual cycle were observed, with a delayed luteal phase and an earlier follicular phase, in a study involving women with PMS (26). Sleep disorders represent a crucial component of PMS symptoms, warranting more comprehensive investigation and specific treatment measures.

### 2.1 Clinical studies

Orexin plays a role in promoting wakefulness, regulates sleep-wake homeostasis, and contributes to circadian rhythm control. Orexin signaling peaks during wakefulness and subsequently decreases during slow-wave and REM sleep (27). PSG studies have demonstrated that orexin-A has a stabilizing effect on nocturnal sleep in a large clinical sample of patients with hypersomnia, and different cerebrospinal fluid (CSF) orexin-A levels correlate with nocturnal sleep/wake rhythms (28). Deficiency, loss, and disruption of signaling in orexin neurons can lead to sleep disorders. Research indicates that patients with reduced CSF orexin-A levels showed shortened mean sleep latency (29) and orexin-A deficiency leads to REM sleep disturbances in patients with narcolepsy (30). Narcolepsy type 1 (NT 1) results from the loss of orexin neurons, leading to low levels of orexin neuropeptides in the brain. In a clinical trial investigating this disorder, Evans et al. found that the OX2R agonist danavorexton significantly improved subjective somnolence in NT 1 participants, dose-dependently increased arousal in NT 1 patients, and effectively treated drowsiness symptoms, although the study noted mild side effects associated with the treatment (31). Suvorexant is a novel orexin receptor antagonist that induces and maintains sleep clinically used in treating insomnia. Herring et al. found that patients treated with suvorexant exhibited greater improvements in subjective and objective PSG measures of sleep onset and sleep maintenance compared to the placebo group in a clinical trial for insomnia treatment. However, in assessing drug safety, patients treated with suvorexant presented mild to moderate somnolence (32). Whether it is suvorexant for insomnia or danavorexton for drowsiness, these findings provide evidence for the role of orexin in the treatment of sleep disorders.

### 2.2 Animal studies

In animal studies, Hung et al. found that the ablation of orexin neurons in the mice brain reduced arousal and resulted in a narcolepsy-like phenotype (33). Murillo-Rodriguez et al. damaged orexin neurons with a neurotoxin in rats and observed that these rats woke up less during the night and had more REM sleep, which is consistent with reduced orexin function (34). Mieda et al. discovered that orexin neuron-ablated mice exhibited narcolepsy, and central administration of orexin-A significantly suppressed cataplectic behavioral arrests and increased wakefulness in these mice (35). Evans et al. (31) found that danavorexton administration increased arousal and reduced arousal fragmentation in orexin/ataxin-3 mice (a narcoleptic mouse model with postnatal hypocretin/orexin cell death) during the active/wakeful period. Furthermore, observations on its pharmacodynamics have informed study design and dose selection for NT 1 cohorts in human studies using intravenous infusion. Additionally, the orexin receptor antagonist suvorexant can promote sleep by blocking OX1R and OX2R (36). Orexin receptor antagonists and agonists provide a novel treatment for sleep disorders, opening new therapeutic avenues in the treatment of PMS/PMDD. The pharmacological role of these drugs in treating sleep disorders in patients with PMS warrants further exploration. These studies offer insights into the treatment of PMS sleep disorders, suggesting that orexin may be a potential target for therapy. However, the distribution and specific mechanisms of orexin and its receptors in PMS/PMDD have not been fully elucidated and may be related to the interaction of orexin with neurotransmitters, such as 5-HT, noradrenaline, acetyl-choline, GABA, and dopamine, etc. Large-scale preclinical and clinical trials are needed to further explore the relevant mechanisms.

## 3 Orexin improved mood and cognitive impairment

### 3.1 Orexin improved depression

Patients with PMS and PMDD are characterized by affective disorders, including anxiety and depression, with depressed mood being the main symptom in patients with PMDD (37). Therefore, alleviating mood symptoms is crucial for PMS. The current drugs of choice for the treatment of PMS/PMDD are first-line antidepressants, namely selective serotonin reuptake inhibitors (SSRIs). However, these drugs’ tolerability and dose-dependent nature of adverse effects limit their widespread clinical use (38). In recent years, research on orexin’s ability to alleviate depressive symptoms has been increasingly recognized and validated in preclinical trials (39). Reduced orexin levels are associated with depressed mood in depressed patients (40). Lu et al. also revealed significant gender-related changes in orexin in the hypothalamus of depressed patients, with female patients showing more pronounced orexin immunoreactivity (41). Research and development of drugs targeting orexin signaling is well established (42, 43) and has shown promise in animal experiments. The Orexin system appears to be a promising target for treating the symptoms of PMS/PMDD.

#### 3.1.1 Clinical studies

In humans, changes in orexin levels in the CSF correlate with physical and emotional changes. Orexin levels in peripheral blood cells of patients with major depression were significantly different from those of healthy patients. It was found that orexin-A mRNA expression levels in patients with major depression were negatively correlated with Hamilton Depression Rating Scale scores (used to assess depressive states) (44). Brundin et al. analyzed 66 patients with major depression who attempted suicide with significantly lower CSF orexin-A levels (45). In a clinical observation, Pu et al. found that the orexin promoter showed 61% methylation in 41 patients with depression with cancer and 43% methylation in 7 patients with depression alone, indicating that higher methylation levels were associated with lower orexin levels and depression (46). It is reported that exercise can increase human blood plasma orexin-A levels (47), and Chieffi et al. concluded that exercise also increases hippocampal volume (48), both of which are relevant to the treatment of depression.

#### 3.1.2 Animal studies

Wistar-Kyoto rats (WKY), a genetic model for depression, exhibit increased depressive-like behaviors, that resemble clinical features found in patients with depression. Compared to normal rats, WKY rats show an 18% reduction in the number of orexin-positive neurons and a 15% reduction in the average soma size of orexin-producing cells in the hypothalamus (49). Reduced orexin-A/OX1R mRNA expression and protein content were observed in the lateral hypothalamic region of a rat model of depression (50), and the chronic unpredictable mild stress (CUMS) model of depression reduced OXR2 expression in the ventral hippocampus, thalamus, as well as hypothalamus of rats (51). Basal levels of orexin-A and orexin-B were reduced in the medial prefrontal cortex, voxel nucleus, ventral tegmental area (VTA), and hypothalamus in the social defeat rat model of depression (52). Furthermore, the elimination of OX1R and OX2R by knockdown or pharmacological blockade significantly induced depressive symptoms in experimental mice (53); In contrast, the OX1R receptor antagonist SB334867 effectively suppressed the orexin-A-induced decrease in immobility duration and increase in bromodeoxyuridine (BrdU)-positive cells (a thymidine analog that labels dividing cells in the S-phase of the cell cycle, which accurately reflects cell proliferation) in the hippocampal dentate gyrus (54). These observations provide clear evidence that orexin is a key target for mood regulation, particularly for alleviating depressed mood. Ito et al. found that orexin enhanced hippocampal dentate gyrus BrdU-positive cells in a dose-dependent manner when orexin-A was injected into the ventricles of mice compared to saline controls, thereby inducing an antidepressant-like effect to a certain extent (54). Similarly, orexin-A injection in the VTA brain region of mice showed depression-like behavior significantly ameliorated depressive symptoms and was found to significantly improve depression-like behavior in behavioral tests in mice through optogenetic and chemogenetic activation of orexin terminals in the VTA of CUMS mice (55). This evidence sheds light on the role of orexin in the pathogenesis of depression and suggests that orexin may be a therapeutic target for depression.

### 3.2 Orexin improved cognitive impairment

In addition to changes in mood, patients with PMDD report impairments in cognitive abilities, including attention, memory, and motor coordination (56). Negative cognitive styles, such as a general negative expectancy for future events and the feeling of having no influence over them, are important factors in the pathogenesis of PMS/PMDD (57). PMDD shares similarities with major depression regarding cognitive vulnerability, and cognitive deficits are well-established in MDD. In a survey of 14 women with PMDD, Reed found that during the luteal phase, women with PMDD exhibited decreased performance on immediate and delayed word recall tasks, immediate and delayed digit recall tasks, and digit symbol replacement tests compared to controls (58). The working memory (WM) system temporally stores and processes information used to guide behavior and includes four subsystems: central execution, visuospatial storage, speech storage, and episode buffering. Yen et al. (59) first demonstrated reduced WM and cognitive control in PMDD patients during premenstrual GO/Nogo tasks, which was relieved during the follicular phase, consistent with abnormal mood and somatic symptoms. Slyepchenko et al. found persistent, subtle WM and selective attention difficulties during the follicular phase of the menstrual cycle in patients with moderate to severe PMS (60). There is also evidence that ovarian hormones are associated with emotional and cognitive processing and affect the cognitive abilities of women with PMDD during the menstrual cycle (61, 62).

Orexin plays an important role in cognitive regulation. It improves cognitive deficits in rats by addressing deficits in neurotransmission (63). Intranasal administration of [Ala11, D-Leu15]-OXB increased c-fos expression (a reliable marker for neural activity) in cortical and basal forebrain areas as well as medial septal cholinergic neurons. Orexin-A also activated a wider range of cortical areas and basal forebrain cholinergic neurons. In addition, intranasal orexin-A significantly reversed the changes produced by sleep deprivation on local brain changes in cerebral metabolic rate for glucose consumption (CMRglc) in rhesus monkeys during delayed match-to-sample task execution more effectively than intravenous injection (64). In orexin/ataxin-3 narcoleptic mice, Yang et al. found that they showed deficits in long-term social memory. Nasal administration of exogenous orexin-A restored social memory to some extent in AT mice, which was associated with enhanced hippocampal synaptic plasticity and cAMP response element binding protein phosphorylation (65). In conclusion, these findings provide strong evidence for the effectiveness of orexin in improving cognitive deficits.

## 4 Orexin regulated ovarian hormone levels

### 4.1 Hormonal fluctuations and PMS

During the menstrual cycle, estrogen levels are low during menstruation, gradually rise during ovulation, and then fall gradually during the luteal phase. On the other hand, progesterone levels remain low throughout the follicular phase, experience a rapid increase after ovulation, and then decrease quickly during the late luteal phase (66). These fluctuations in hormone levels may significantly contribute to mood swings experienced by women. Numerous studies have underscored the impact of estrogen and progesterone on depression, anxiety, and other mood disorders in women, implicating abnormal hormone fluctuations as crucial mechanisms in the development of premenstrual depression (67). Different from healthy women, those with PMS exhibit lower estrogen levels and higher progesterone levels. It has been found that the rapid withdrawal of ovarian hormones is likely pivotal in the pathogenesis of PMDD (68). Women with PMDD have demonstrated lower early luteal estrogen levels compared to controls, and covariate analysis demonstrates that the interaction between early luteal phase estrogen and progesterone levels correlates with PMDD severity (69). Both preclinical and human research have illustrated that the cessation of ovarian steroids (estrogen, progesterone, or both) can trigger emotional, cognitive, and behavioral symptoms associated with suicide in susceptible individuals (70). The link between allelic variants in the estrogen receptor alpha gene and PMDD demonstrated by Huo et al. was the first finding revealing genetic alteration in the background of this disorder. ERα’s involvement in the etiopathogenesis and treatment of PMDD, through its regulation of neurotransmitter system signal transduction, underscores its potential significance (71). In addition, Pakharenko et al. have verified that polymorphic variants in the estrogen receptor 1 (ESR1) A-351G gene can serve as markers for the development of PMS (72). Intriguingly, estrogen-mediated signaling also plays a significant role in the activation of neural circuits associated with social cognition and emotion regulation (62). In conclusion, these studies demonstrate the relevance of ovarian hormones to PMS/PMDD. However, it is important to acknowledge that the production of hormones and their actions in the body involve complex molecular mechanisms; consequently, ovarian hormone therapy is not merely about controlling estrogen and progesterone levels, necessitating further comprehensive investigations.

### 4.2 Orexin regulated steroid hormones

The transcription and translation of prepro-orexin mRNA take place in the hypothalamic region, a crucial locus for the generation of orexin neurons and the primary origin of the hypothalamic-pituitary-gonadal axis (HPG). Consequently, it is plausible that the regulation of behavioral and stress responses in the hypothalamus may be intricately linked to the coordinated interactions among the E2, progesterone, and orexin systems (73). Orexin neurons play an essential role in the regulation of the HPG axis and sexual behavior (74, 75). Research by Cataldi et al. indicates that both orexin-A and orexin-B diminish progesterone secretion in luteal cells, suggesting a modulatory role for the orexin system within the ovary (76). Notably, both *in vivo* and *in vitro* investigations have revealed that orexin-A inhibits luteinizing hormone-releasing hormone (LHRH)-stimulated LH release in the dispersed anterior pituitary of proestrus female rats in a dose-dependent manner (75). The hormonal secretion during the rodent estrous cycle appears to correlate with appropriate nutritional status and alertness. It has been demonstrated that orexin governs luteinizing hormone-releasing steroids, nutritional status, and alertness in an interdependent fashion, thus hinting at a possible coordinating function for orexin throughout the estrous cycle (77). Similarly, Kiezun et al. explored alterations in the estrous cycle and early pregnancy in the pig uterus concerning estrogen E1 and E2 emphasizing the orexin system’s role as a “molecular switch” for estrogen activation (78). In conclusion, this body of evidence suggests that orexin may have therapeutic effects in PMS/PMDD through its modulation of ovarian hormones. However, for PMS, there exists inconsistency in the results pertaining to hormone expression levels despite the increasing recognition of the trend toward lower estrogen levels and higher progesterone. Furthermore, the molecular activity and downstream signaling pathways of orexin in the context of intricate hormonal mechanisms remain largely unelucidated, necessitating comprehensive mechanistic studies.

## 5 Orexin regulated the neuroimmune system to inhibit the inflammatory response

### 5.1 Inflammatory mechanisms in PMS/PMDD

Emerging trends in neuroendocrinology indicate a connection between inflammatory processes and both mental and somatic disorders sharing common features with PMS/PMDD. Low-level inflammation has been associated with alterations in mood and pain. In a study investigating the correlation between changes in physical and psychological symptoms and inflammatory markers during the menstrual cycle in normal weight and overweight women. Puder et al. discovered that variations in tumor necrosis factor-α (TNF-α) and high-sensitivity C-reactive protein serum concentrations were associated with physical and psychological symptoms experienced during the menstrual cycle (79). Another investigation focusing on the association between inflammatory markers and the severity of menstrual symptoms and PMS among young women revealed that mean levels of interleukin 4, interleukin 10 (IL-10), interleukin 12, and Interferon-gamma were significantly elevated in women meeting PMS criteria compared to healthy women (80). In a comprehensive review by Hantsoo et al. on the epidemiology and treatment of PMS, it was suggested that inflammatory molecules may contribute to the pathobiology of PMDD (81). Furthermore, in a survey involving 21 healthy women with regular menstrual cycles, plasma IL-10 levels were notably higher in women experiencing PMS compared to their healthy counterparts (82). Cumulatively, these findings provide substantial preliminary evidence supporting a potential link between neuroinflammation and the etiology of PMS (83).

### 5.2 Orexin inhibited inflammatory response to protect neurons

Orexin neurons play an important role in neuroprotection, primarily through the inhibition of inflammatory responses mediated by OX1R and OX2R (84). Experimental findings demonstrated that diminished orexin signaling plays a crucial role in mediating inflammation-induced drowsiness (85). Clark et al. proposed that the activity of orexin neurons is suppressed by the inflammatory cytokine TNF, which has the capacity to degrade orexin precursor mRNA in a time- and dose-dependent manner (86). In a study by Modi et al., utilizing intranasal orexin in a rat model of cardiac arrest (CA), it was revealed that CA led to increased pro-inflammatory markers across all brain regions. Treatment with orexin-A significantly ameliorated CA-induced neuroinflammatory markers in the hypothalamus, increased the production of OX1R and OX2R, exerted anti-inflammatory effects, and expedited cortical EEG and behavioral recovery (87). Additionally, Zhang et al. found that orexin-A effectively dampened endothelial cell inflammation by inhibiting mitogen-activated protein kinases p38 and nuclear factor kappa-B (NF-κB) inflammatory signaling pathways (88). In an investigation into the molecular mechanisms by which orexin-A reduces cerebral ischemia/reperfusion injury, Xu et al. observed that orexin-A confers neuroprotective effects by inhibiting OX1R-mediated NF-κB, mitogen-activated protein kinase (MAPK)/extracellular signal-regulated kinase, and MAPK/p38 signaling pathways, and by suppressing astrocyte apoptosis, activation, and the generation of inflammatory factors such as interleukin 1β (IL-1β), TNF-α, and interleukin 6 (IL-6) (89). Moreover, examining the physiological function of orexin-A in fibroblast-like synoviocytes, Sun et al. found that orexin-A treatment resulted in reduced secretion of IL-1β, IL-6, and interleukin 8, decreased production of reactive oxygen species, and inhibited TNF-α-induced activation of the NF-κB signaling pathway, thus demonstrating an important role in neuroprotection (84). This body of evidence reveals the antagonistic relationship between orexin and inflammatory cytokines, underscoring the potential of orexin’s anti-inflammatory effect as a promising new target for the treatment of PMS/PMDD.

## 6 Modulation of the GABA system by orexin

### 6.1 Involvement of the GABA system in the etiology of PMS

Gamma-aminobutyric acid (GABA) serves as the predominant inhibitory neurotransmitter in the human cortex and plays a pivotal role in regulating salience plasticity, sleep, and mood (90–92). Patients diagnosed with PMS often demonstrate alterations in GABA-α receptor sensitivity and GABA concentration, exhibiting reduced plasma GABA levels during the late luteal phase, particularly in cases of premenstrual anxiety (93). Fluctuations in steroid hormone levels can influence the structure and function of the GABAA receptor (GABAAR), impacting the expression levels of GABAARγ-2 and delta subunits, which are closely associated with the pathophysiology of PMS (94, 95). In a study by Zhang et al., analysis of GABA receptor subunit expression patterns in a rat model simulating clinical PMDD symptoms revealed notable differences in the expression of GABAAR α1, α2, α4, α5, β2, β3, and δ subunits among PMDD rat models with distinct characteristics, suggesting their potential as biomarkers for PMDD pathogenesis (96). Furthermore, experimental findings by Iba et al. indicated that the Japanese herb Inochinohaha White increased the b2-subunit of the GABAA receptor in the amygdala, attenuating anxiety-like behavior in a rat model of progesterone withdrawal through augmentation of the GABAA receptor-mediated signaling pathway (97). Similarly, the Chinese medicinal preparation Shuyu capsule, utilized for treating PMS and comprising active ingredients extracted from four Chinese medicines (bupleurum chinense, white paeony root, radix glycyrrhizae, and rhizoma cyperi), reversed the decrease in glutamate to GABA ratio in the right hippocampus of a rat model of premenstrual depression (98). These investigations provide encouraging evidence for the relationship between the GABA system and PMS, warranting further exploration of GABA as an effective therapeutic target for PMS.

### 6.2 Orexin regulates PMS symptoms through the GABA system

GABA and glutamate neurons are distributed in the reticular core of the brain, where they play a crucial role in regulating cortical activity and behavior during the wake-sleep state. These in turn are modulated by acetylcholine, noradrenaline, dopamine, serotonin, histamine, orexin, and the melanin-concentrating hormone neurons (99). The interplay between orexin and GABA neurotransmission is particularly significant in sleep and mood regulation. Ji et al. found that orexin neurons have direct projections to GABA neurons in the ventral pallidum, enabling them to modulate GABA depolarization via OX1R and OX2R. Knockdown or pharmacological blockade of orexin receptors led to a decrease in peak current in GABA neuron cells. Furthermore, exogenous orexin was found to activate GABA neurons via OX1R and OX2R thereby ameliorating depressive-like behavior in rats (46). Moreover, postsynaptic OX2R activation has been shown to stimulate Na/Ca2 exchange currents in GABA neurons, resulting in heightened membrane depolarization, increased firing rates, and augmented GABA release (100). Similarly, Brevig et al. demonstrated that intracerebral pontine reticular formation injection of orexin-A in rats led to an increase in consolidated wakefulness through the activation of GABAA receptors and OX1R, ultimately promoting arousal (101). Orexin has been found to enhance neuronal plasticity via its modulation of GABA neuron release, exerting selective influence on sleep/wake control within the brain (102), and contributing to the regulation of mood changes. This evidence suggests that the activation of GABA by orexin may represent a potential target for the treatment of PMS/PMDD.

## 7 Conclusion

In summary, orexin may play a pivotal role in the pathomechanism of PMS, thus showing therapeutic potential to ameliorate symptoms of PMS. Orexin improves sleep disorder, suppresses depression, and improves cognitive deficits, all of which are crucial components of the pathologic expression of PMS/PMDD. Additionally, orexin may also be beneficial in improving hormonal abnormalities, inhibiting neuroinflammation, and modulating GABA neurotransmission, as well as it exerts neuroprotective effects. Therefore, orexin may be a potential target for PMS/PMDD treatment.

The physiological effects of orexin in PMS/PMDD remain unexplored and orexin receptor antagonists have not been used in the clinical treatment of PMS/PMDD. Research on the use of orexin receptor antagonists or agonists in sleep disorders presents promising potential as a therapeutic agent, warranting further investigation and exploration. This review laid the groundwork for future studies examining orexin’s role and application in PMS. Ultimately, orexin holds promise as a therapeutic target for PMS, and the development of related drugs may offer novel treatments for PMS/PMDD in the future, through direct or indirect modulation of the orexin system.

## Author contributions

PD: Writing – original draft. WD: Writing – original draft. MS: Writing – original draft. SW: Writing – original draft. YM: Writing – review & editing. TZ: Writing – review & editing. FZ: Writing – review & editing. PS: Writing – review & editing.
